# Decreased B4GALT1 promotes hepatocellular carcinoma cell invasiveness by regulating the laminin-integrin pathway

**DOI:** 10.1038/s41389-023-00494-y

**Published:** 2023-10-31

**Authors:** Po-Da Chen, Ying-Yu Liao, Yu-Chia Cheng, Hsin-Yi Wu, Yao-Ming Wu, Min-Chuan Huang

**Affiliations:** 1 Graduate Institute of Anatomy and Cell Biology, College of Medicine, National Taiwan University, Taipei, Taiwan; 2 Department of Surgery, National Taiwan University Hospital, Taipei, Taiwan; 3 Department of Surgical Oncology, National Taiwan University Cancer Center, Taipei, Taiwan; 4 Instrumentation center, National Taiwan University, Taipei, Taiwan

**Keywords:** Liver cancer, Extracellular matrix

## Abstract

Beta1,4-galactosyltransferases (B4GALTs) play a crucial role in several diseases, including cancer. B4GALT1 is highly expressed in the liver, and patients with mutations in *B4GALT1* exhibit hepatopathy. However, the role of B4GALT1 in liver cancer remains unclear. Here, we found that B4GALT1 was significantly downregulated in hepatocellular carcinoma (HCC) tissue compared with the adjacent liver tissue, and low B4GALT1 expression was associated with vascular invasion and poor overall survival in patients with HCC. Additionally, silencing or loss of B4GALT1 enhanced HCC cell migration and invasion in vitro and promoted lung metastasis of HCC in NOD/SCID mice. Moreover, B4GALT1 knockdown or knockout increased cell adhesion to laminin, whereas B4GALT1 overexpression decreased the adhesion. Through a mass spectrometry-based approach and *Griffonia simplicifolia* lectin II (GSL-II) pull-down assays, we identified integrins α6 and β1 as the main protein substrates of B4GALT1 and their N-glycans were modified by B4GALT1. Further, the increased cell migration and invasion induced by B4GALT1 knockdown or knockout were significantly reversed using a blocking antibody against integrin α6 or integrin β1. These results suggest that B4GALT1 downregulation alters N-glycosylation and enhances the laminin-binding activity of integrin α6 and integrin β1 to promote invasiveness of HCC cells. Our findings provide novel insights into the role of B4GALT1 in HCC metastasis and highlight targeting the laminin-integrin axis as a potential therapeutic strategy for HCC with low B4GALT1 expression.

## Introduction

Hepatocellular carcinoma (HCC) is the most prevalent liver malignancy and the leading cause of liver cancer-related deaths worldwide [1]. This high mortality rate is due to the fact that the disease is often diagnosed at the advanced stage, including vascular invasion or metastatic disease [2]. Thus, approximately 80% of patients with HCC do not undergo curative treatment [3]. In validating modern prognostic models, vascular invasion is mostly included in the proposed factors and has increasingly been adopted to reflect the risk of local invasion and distant metastasis [4, 5]. Considering the vascular invasion has emerged as a key contributor to tumor progression, efforts are devoted to elucidate the molecular mechanisms of cell invasion through the extracellular matrix (ECM) in HCC [6].

In the context of HCC progression, ECM remodeling has been detected in the tumor microenvironment [7]. Integrins are the largest family of ECM receptors. Previous studies indicated that several integrins and matrix-binding proteins are notably increased in HCC tissue compared with their levels in normal liver tissue [8, 9]. Studies have also shown that integrins and the associated ECM mediate HCC cell chemotaxis are associated with poor prognosis and worse malignancy [10, 11]. Glycosylation has been repeatedly demonstrated to tightly regulate the functions of integrins [12]. To target integrin-regulated malignancies, it will be of great importance to thoroughly understand how glycosylation modulates the activity of integrins.

There is emerging evidence that abnormal oligosaccharide expression is associated with HCC [13, 14]. The necessity of strict regulation regarding the extending, elongating, and branching of glycans maintains the normal cell function; on the contrary, altered or dysregulated processing of glycan structures has been recognized as a contributing factor to cancer [15, 16]. It is suggested that cell migration and invasion reflect metastasis in clinical conditions and disease survival; and dysregulated glycosylation plays a pivotal role in the invasive behaviors by altering the tumor cell-microenvironment interaction [17]. Beta-1,4-galactosyltransferases (B4GALTs) are a family of evolutionally conserved enzymes that transfer galactose (Gal) to N-acetylglucosamine (GlcNAc) acceptor sugars in a beta-1,4 linkage [18]. The diverse glycans generated play a wide range of biological functions, including protein folding, host-pathogen interaction, immune rescancerponse [19], regulation of growth factor distribution, and cell adhesion. Among 7 members, B4GALT1 is considered the most important enzyme for the galactosylation of N-glycans [20]. Mice with *B4galt1* knockout were mainly characterized by growth retardation and early death [21]. Moreover, platelets in mice lacking *B4galt1* adhered avidly to β1 integrin ligands laminin, fibronectin, and collagen [22]. Northern blot analysis indicated that human *B4GALT1* mRNA is highly expressed in the liver [23]. It has been reported that the phenotype of patients with mutation in *B4GALT1* includes inherited coagulation disturbances with hepatopathy and hepatomegaly [24]. Several lines of evidence indicate that high B4GALT1 expression is associated with poor survival of patients and promotes malignant behaviors in several cancer types, such as lung cancer [25], bladder cancer [26], pancreatic ductal adenocarcinoma [27], renal cancer [28], glioblastoma [29], leukemia [30], etc. Despite important biological functions of galactosylation and high expression of *B4GALT1* in the liver, the role of B4GALT1 in HCC still remains elusive.

In this study, analyses of clinical specimens revealed that B4GALT1 was downregulated in HCC and low B4GALT1 expression was associated with poor overall survival of patients with HCC. Moreover, decreased B4GALT1 expression promoted invasiveness of HCC cells in vitro and in vivo. Mechanistically, we used an unbiased proteomic approach to identify B4GALT1 protein substrates and uncovered a critical role of B4GALT1 in HCC invasiveness through modulation of activity and galactosylation of the laminin receptor integrin α6β1.

## Results

### Low B4GALT1 expression is associated with poor survival in patients with HCC

**Fig. 1:**
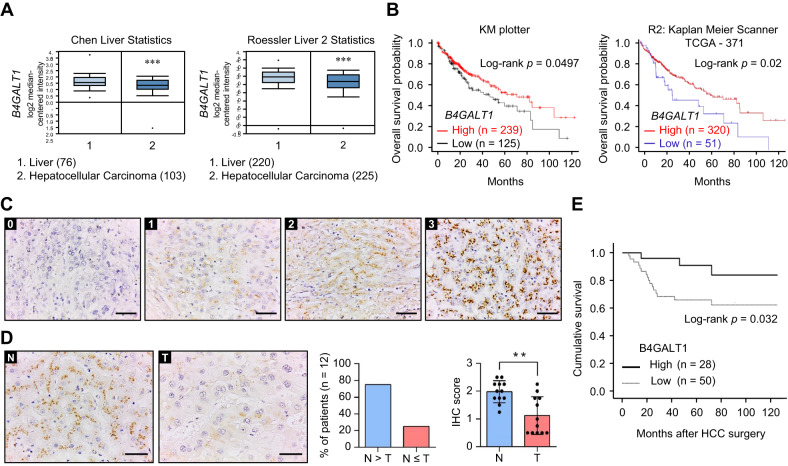
Clinical significance of B4GALT1 expression in HCC cells. **A** Data from Oncomine indicated the downregulation of B4GALT1 expression in HCC tissue compared with normal liver tissue. Two datasets, Chen Liver and Roessler Liver 2, were analyzed. ***P < 0.001. **B** Kaplan-Meier (KM) survival analysis for patients with HCC based on the KM plotter and R2 (Genomics Analysis and Visualization Platform) webtools. B4GALT1 in HCC tumors were categorized as low and high expression as indicated. *P* value was calculated using the log-rank test. **C** Scoring of B4GALT1 expression determined via immunohistochemistry (IHC). Scale bar, 50 µm. **D** B4GALT1 expression in HCC tumor (T) and its paired non-tumor liver tissue (N). N > T indicates that B4GALT1 expression in the non-tumor liver tissue is higher than that in the HCC tumor. N ≤ T indicates that B4GALT1 expression in the non-tumor liver tissue is less than or equal to that in the HCC tumor. Representative images are shown. Scale bar, 50 µm. **E** Kaplan-Meier survival analysis of B4GALT1 expression in HCC tissue from patients. *P* value was calculated using the log-rank test. Data are represented as mean ± SD. **P < 0.01, Student’s *t*-test.

According to data in The Human Protein Atlas (TCGA) database, among B4GALT family members, B4GALT1 shows the highest expression level in the liver (Supplementary Fig. S1A). Moreover, it is also highly expressed in the liver compared to other organs (Supplementary Fig. S1B). Data from the Chen liver [31] and Roessler liver [32] datasets of Oncomine database revealed that *B4GALT1* mRNA expression is lower in the HCC tissue than normal liver tissue (Fig. 1A). In addition, the TCGA dataset analyzed using the UALCAN platform showed that there are no significant differences in *B4GALT1* mRNA expression between normal liver and hepatocellular carcinoma (HCC) tissues (Supplementary Fig. S2A). According to the TCGA dataset and the Llovet-91 dataset from the R2 platform, the expression of *B4GALT1* is not significantly associated with HCC stages (Supplementary Fig. S2B). Survival analysis using KM plotter and R2 platforms showed that low *B4GALT1* mRNA expression is associated with poor overall survival for patients with HCC (Fig. 1B). Further, in this study, we analyzed B4GALT1 expression in HCC tissue sections as well as its correlation with the clinicopathological features and prognosis of HCC. Based on the immunohistochemical staining intensity, we scored B4GALT1 expression from 0 to 3 (Fig. 1C), with scores 0–1 and 2–3 classified as low and high B4GALT1 expression, respectively. The results confirmed lower B4GALT1 expression in HCC tissue relative to adjacent non-tumor liver tissue (Fig. 1D). We analyzed the scRNA-Seq profile of HCC and stromal cells (dataset ID: EGAD00001006190). The tSNE plots showed that there are eight major cell types in HCC (Supplementary Fig. S2C, left panel). The *B4GALT1* mRNA level was shown by differential degrees of blue color (Supplementary Fig. S2C, middle panel). The results indicated that *B4GALT1* is expressed in all major cell types, including hepatocellular carcinoma (HCC), myeloid, T, NK, B, plasma, endothelial, and fibroblast cells at variable levels (Supplementary Fig. S2C, right panel). In addition, our immunohistochemical results showed that the stromal cells, including endothelial cells, fibroblasts, and cholangiocytes, in normal liver expressed low levels of B4GALT1 (Supplementary Fig. S3A). The matched control IgG did not show any specific staining in liver (Supplementary Fig. S3B). Moreover, Western blot analysis indicated that B4GALT1 could be easily detected in HCC cell lines and was expressed in different cancer cell lines at variable levels (Supplementary Fig. S3C). Clinicopathological features were determined according to the following criteria: TNM staging was based on the American Joint Committee on Cancer system, vascular invasion was based on the microvascular invasion of tumor cells, and the differentiation grade of the disease was based on the ES grading system. Our results indicated that low B4GALT1 expression was correlated with a high vascular invasion rate (Table 1). Further, Kaplan-Meier survival curves indicated low B4GALT1 expression was associated with poor overall survival (*p* = 0.032; Fig. 1E). Taken together, our results suggest that B4GALT1 is significantly downregulated in HCC tissue compared with its adjacent non-tumor tissue and that low B4GALT1 expression is associated with poor overall survival of patients with HCC.

**Table 1:** Correlation of B4GALT1 intensity and clinicopathologic features.

	High expression (n = 28)	Low expression (n = 50)	*P* value
Sex Male:Female	20:8	27:23	0.569
Cirrhosis	12	17	0.656
Tumor size (cm^3^)	83.6 (2–638)	178.1 (2–1320)	0.172
Encapsuled	12	25	0.303
Vascular invasion	4	24	0.004**
Metastasis	1	1	0.281
Differentiation grade (1:2:3:4)	1:12:14:1	0:25:23:2	0.365
TNM Staging(I:II:III:IV)	19:4:4:1	21:19:9:2	0.217
Recurrence	10	14	0.412
Overall survival (months)	70.4 (15–125)	54.9 (5–125)	0.032*

*P < 0.05; **P < 0.01.

### B4GALT1 regulates the malignant behaviors of HCC cells

**Fig. 2:**
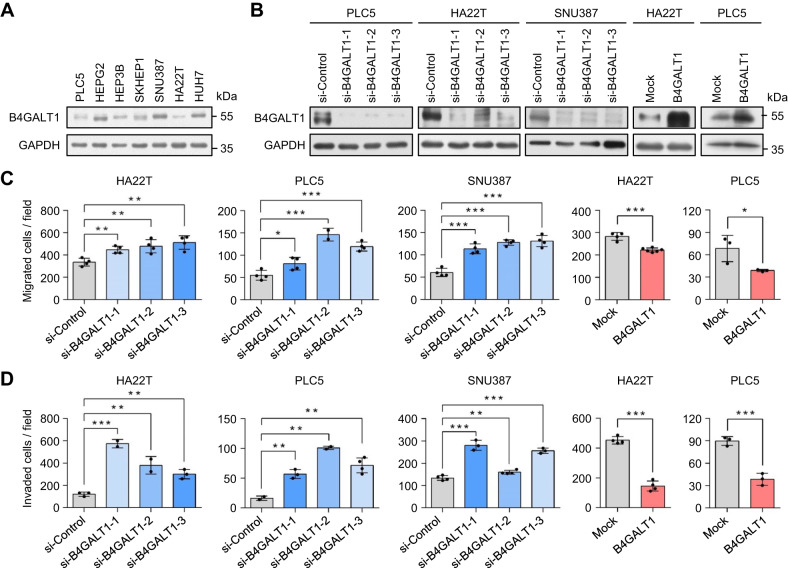
B4GALT1 regulates the malignant behaviors of HCC cells in vitro. **A** Western blots showing B4GALT1 expression in HCC cell lines. **B** Western blots showing B4GALT1knockdown and overexpression in HCC cells. Three independent siRNAs against B4GALT1 (si-B4GALT1-1, si-B4GALT1-2, and si-B4GALT1-3) were used for B4GALT1 knockdown in PLC5, HA22T, and SNU387 cells. Non-targeting siRNA (si-Control) was used as control. For the stable overexpression of B4GALT1 in PLC5 and HA22T cell lines, the B4GALT1-pcDNA3.1 (B4GALT1) plasmid was used. The empty pcDNA3.1 plasmid (mock) was used as the control. **C** Cell migration analyzed via transwell migration assay. **D** Cell invasion analyzed via Matrigel invasion assay. Representative results from three independent experiments are shown. Data are presented as the mean ± SD. *P < 0.05; **P < 0.01; ***P < 0.001, Student’s *t-*test.

To assess the effect of B4GALT1 on HCC cells, we analyzed PLC5, HA22T, SNU387 cell viability, migration, and invasion via MTT, transwell migration, and Matrigel invasion assays, respectively. First, we analyzed B4GALT1 levels in a panel of HCC cell lines and the results showed variable B4GALT1 expression in HCC cells (Fig. 2A). To analyze the effect of B4GALT1 on HCC cells, knockdown and overexpression of B4GALT1 in PLC5, HA22T, and SNU387 cells were performed and confirmed via western blot analysis (Fig. 2B). MTT assays showed that B4GALT1 knockdown slightly increased HA22T cell viability, but decreased PLC5 and SNU387 cell viability (Supplementary Fig. S4). Moreover, B4GALT1 overexpression did not show significant effects on PLC5 and HA22T cell viability. These results suggest that that there was no consistent effect of B4GALT1 on viability of HCC cells. Interestingly, B4GALT1 knockdown in HA22T, PLC5, and SNU387 cells enhanced cell migration and invasion, whereas its overexpression in HA22T and PLC5 cells inhibited cell migration and invasion (Fig. 2C-D). Taken together, these results suggest that low B4GALT1 expression promotes the invasive behaviors of HCC cells, which is consistent with the correlation of low B4GALT1 expression with high vascular invasion in the clinicopathological analysis.

### Effects of B4GALT1 on metastasis of HCC cells in NOD/SCID mice

**Fig. 3:**
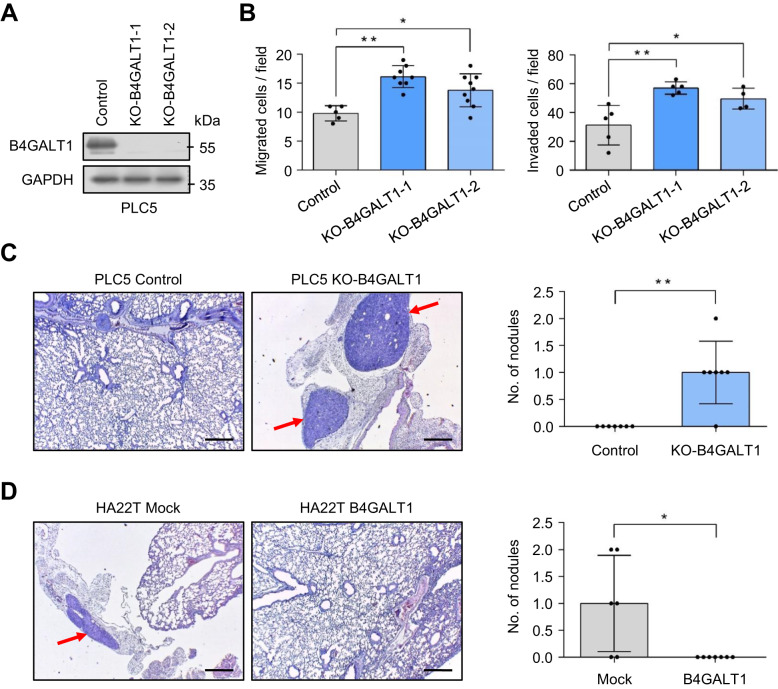
B4GALT1 regulates HCC cell metastasis in vivo. **A** Western blots showing B4GALT1 knockout (ko-B4GALT1-1 and ko-B4GALT1-2) in PLC5 cells, and B4GALT1 overexpression (B4GALT1) in HA22T cells. **B** Effects of B4GALT1 knockout on the migration and invasion of PLC5 cells analyzed via transwell migration and Matrigel invasion assays, respectively. Representative results from three independent experiments are shown. **C** Effects of B4GALT1 knockout on tumor metastasis. Representative images of lung metastasis in parental and B4GALT1 knockout PLC5 cells are shown. The arrows indicate tumor nodules. Scale bar, 500 µm. PLC5 cells were intravenously injected into NOD/SCID mice (*n* = 7 per group) and after 60 days, the mice were sacrificed and their lungs were visualized. **D** Effects of B4GALT1 overexpression on tumor metastasis. Representative images of lung metastasis of HCC in mock and B4GALT1 overexpressing HA22T cells; *n* = 7 per group; one mouse in the mock control group died during the experiment. Data are presented as the mean ± SD. *P < 0.05; **P < 0.01, Student’s t-test.

To further investigate the effect of B4GALT1 on the invasive behavior in vivo, B4GALT1 knockout PLC5 cells or B4GALT1 overexpressing HA22T cells were injected into the tail vein of NOD/SCID mice and the tumor nodule formation in the lungs was also evaluated. B4GALT1 knockout with CRISPR/Cas9 system in PLC5 cells was confirmed using western blot analysis (Fig. 3A). The two independent clones of B4GALT1 knockout PLC5 cells showed enhanced cell migration and invasion (Fig. 3B), consistent with the phenotype of B4GALT1 knockdown in PLC5 cells. In experimental metastatic model, B4GALT1 knockout enhanced lung metastasis of PLC5 cells (Fig. 3C). In contrast, B4GALT1 overexpression inhibited lung metastasis of HA22T cells. These findings suggest that loss of B4GALT1 enhances lung metastasis of HCC cells, while B4GALT1 overexpression suppresses the metastasis in vivo.

### Identification of integrins β1 and α6 as B4GALT1 protein substrates using mass spectrometry

**Fig. 4:**
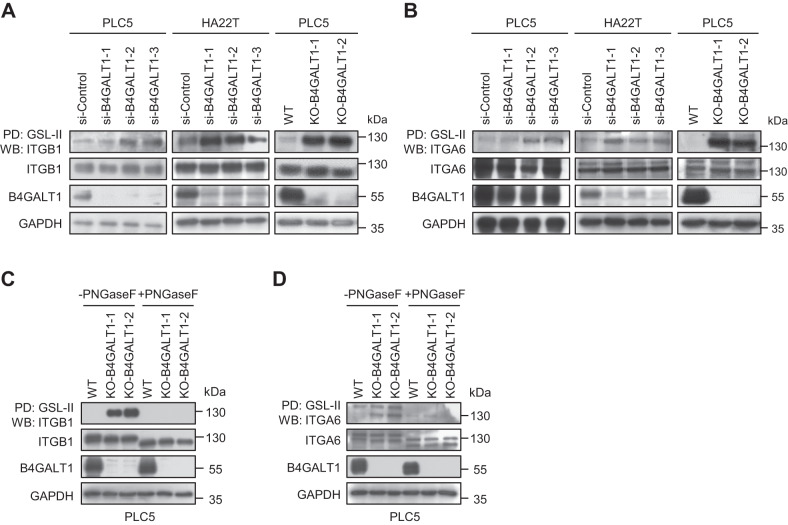
B4GALT1 regulates the glycosylation of integrins α6 and β1 in HCC cells. **A** GSL-II lectin pull-down assay for integrin β1. Proteins in cell lysates from B4GALT1 knockdown PLC5 and HA22T cells and B4GALT1 knockout PLC5 cells were pulled down using GSL-II agarose beads and immunoblotted using an anti-integrin β1 (ITGB1). Three independent siRNAs for B4GALT1and two independent B4GALT1 knockout clones were used, as indicated. **B** GSL-II lectin pull-down assay for integrin α6. Proteins in the cell lysates from B4GALT1 knockdown PLC5 and HA22T cells and B4GALT1 knockout PLC5 cells were pulled down using GSL-II agarose beads and immunoblotted using an anti-integrin α6 (ITGA6). **C** B4GALT1 predominantly modified N-glycans on integrin β1. PLC5 cell lysates (0.5 mg) were treated with PNGase F and then pulled down (PD) using GSL-II lectin. The pull-down proteins were separated using 6% SDS-PAGE and analyzed via immunoblotting with anti-ITGB1. **D** B4GALT1 predominantly modified N-glycans on integrin α6. PLC5 cell lysates (0.5 mg) were treated with PNGase F and then pulled down (PD) using GSL-II lectin. The pulled-down proteins were separated using 6% SDS-PAGE and analyzed via immunoblotting using anti-ITGA6 (A) or anti-ITGA6 (B) antibody. Representative results from three independent experiments are shown. Data are presented as the mean ± SD. *P < 0.05; **P < 0.01; ***P < 0.001, Student’s *t*-test.

To investigate the effects of B4GALT1 on glycophenotypes, flow cytometry of B4GALT1 knockout PLC5 cells with a series of lectins was performed. As expected, the results showed that loss of B4GALT1 dramatically increased GSL-II binding to PLC5 cells (Supplementary Fig. S5). In contrast, LEL, RCA-I, ECL, MAL, and PNA did not show significant changes. This finding suggests that B4GALT1 knockout inhibited the addition of Gal to GlcNAc and therefore exposed the GlcNAc on the complex type of N-glycans on cell surfaces. Next, to identify B4GALT1 protein substrates, we pulled down proteins with GSL-II agarose in wild type and B4GALT1 knockout PLC5 cells and then performed mass spectrometry (MS). The MS data revealed 10 proteins with at least a 2-fold change in GSL-II binding (Supplementary Table S1), which are potential B4GALT1 protein substrates. Among them, the function of integrins β1 (ITGB1) and α6 (ITGA6) is highly correlated with cell invasion [33, 34]. We therefore hypothesized that B4GALT1 may regulate HCC cell invasion through integrins β1 and α6. First, we assessed the surface expression of integrins β1 and α6 in HCC cells using flow cytometry and found that both integrins β1 and α6 were expressed on PLC5, HA22T, SNU387, and HepG2 cells (Supplementary Fig. S6). To validate MS data, GSL-II lectin pull-down assays of integrins β1 and α6 were performed in B4GALT1 knockdown PLC5 and HA22T cells as well as B4GALT1 knockout PLC5 cells. The results revealed that the downregulation of B4GALT1 enhanced GSL-II binding to integrins β1 and α6 (Fig. 4A, B). Moreover, B4GALT1 knockout dramatically increased the binding, confirming the modification of integrins β1 and α6 by B4GALT1. To determine whether B4GALT1 regulates O-glycans or N-glycans on integrins β1 and α6, PLC5 cell lysates were treated with PNGase F and then pulled down using GSL-II lectin. The results showed that the removal of N-glycans by PNGase F treatment decreased the molecular weight of integrins β1 and α6. Moreover, the increased GSL-II binding to integrins β1 and α6 caused by B4GALT1 knockout was almost completely blocked (Fig. 4C, D), suggesting that B4GALT1 predominantly modified the N-glycans of integrins β1 and α6 in HCC cells. Taken together, these results suggest that N-glycans on integrins β1 and α6 are modified by B4GALT1 in HCC cells and imply that integrins β1 and α6 are involved in HCC cell invasion modulated by B4GALT1.

### Effects of B4GALT1 on HCC cell-extracellular matrix (ECM) adhesion

**Fig. 5:**
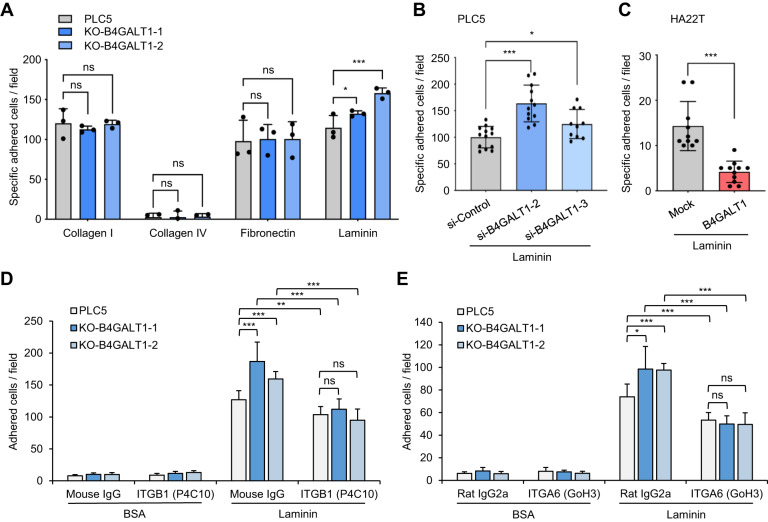
B4GALT1 regulates HCC cell-laminin adhesion. **A** Effects of B4GALT1 knockout on cell-ECM adhesion. Wild-type (control) and B4GALT1 knockout PLC5 cells (two clones: ko-B4GALT1-1 and ko-B4GALT1-2) were seeded into 96-well plates coated with collagen I, collagen IV, fibronectin, or laminin. After incubation for 30 min, the wells were washed three times with PBS and the number of adhered cells was counted under an inverted microscope. Representative results from three independent experiments are shown. The number of specific adhered cells is calculated by the number of cells adhered on ECM subtracted by those adhered to BSA. **B** Effects of B4GALT1 overexpression on cell-laminin adhesion in HA22T cells. Mock and B4GALT1 overexpressing HA22T cells were seeded into 96-well plates coated with laminin. **C** Effects of B4GALT1 knockdown on cell-laminin adhesion in PLC5 cells. Control and B4GALT1 knockdown PLC5 cells were seeded into 96-well plates coated with laminin. Two independent siRNAs (si-B4GALT1-2 and si-B4GALT1-3) were used. **D** Wild-type (WT) or B4GALT1 knockout PLC5 cells were pre-treated with integrin β1 blocking antibody (P4C10) or mouse IgG control for 10 min and then plated into laminin-coated 96 well plates for 30 min. **E** WT and B4GALT1 KO cells were pre-treated with integrin α6 blocking antibody (GoH3) for 10 min and then plated into laminin-coated 96 well plates for 30 min. Rat IgG2a was used for control. Data are presented as the mean ± SD. *P < 0.05; **P < 0.01; ***P < 0.001; ns, not significant. Student’s *t*-test.

Cell-ECM adhesion plays a crucial role in cell migration and invasion. Next, we investigated whether B4GALT1 can regulate cell-ECM adhesion by plating control or B4GALT1 knockout PLC5 cells to 96-well dishes coated with collagen I, collagen IV, fibronectin, and laminin in serum-free medium. We observed that B4GALT1 knockout significantly enhanced cell adhesion to laminin but not collagen I, collagen IV, and fibronectin in PLC5 cells (Fig. 5A). To further confirm the effect of B4GALT1 on laminin binding, we performed cell-laminin adhesion assays using B4GALT1 overexpressing HA22T cells and B4GALT1 knockdown PLC5 cells. We found that B4GALT1 overexpression inhibited cell adhesion to laminin in HA22T cells (Fig. 5B). In contrast, B4GALT1 knockdown enhanced the cell-laminin adhesion in PLC5 cells (Fig. 5C). To further confirm the role of integrins β1 and α6 in cell-laminin adhesion, we analyzed whether integrin-specific blocking antibodies can affect cell adhesion to laminin. The results showed that the increased cell-laminin adhesion by B4GALT1 knockout was significantly reversed by the blocking antibody against integrin β1 (Fig. 5D) or integrin α6 (Fig. 5E) in PLC5 cells. These findings suggest that low B4GALT1 expression enhances HCC cell adhesion to laminin in HCC cells and imply that the activity of integrin α6β1, a laminin receptor, on HCC cells is modulated by B4GALT1.

To know whether the presence of GlcNAc plays a role in the integrin-mediated cell-laminin adhesion, we preincubated PLC5 cells with various lectins or removed GlcNAc on PLC5 cells with N-acetylglucosaminidase and then performed cell-laminin adhesion assay. We found that GlcNAc-binding lectin GSL-II was able to inhibit cell adhesion to laminin in B4GALT1 knockout but not wild-type PLC5 cells (Supplementary Fig. S7). Moreover, RCA-I, a galactose (Gal) binding lectin, almost completely blocked cell-laminin adhesion, indicating that Gal residues could be highly decorated on integrins. By contrast, VVA, a GalNAc binding lectin, did not show any effect. After removal of GlcNAc, the increased adhesion by B4GALT1 knockout was reversed (Supplementary Fig. S8A). Flow cytometry of PLC5 cells with GSL-II confirmed the GlcNAc removal with N-acetylglucosaminidase (Supplementary Fig. S8B). These results suggest that GlcNAc residues are decorated on integrins and may contribute to cell-laminin adhesion.

Sialic acids are often decorated on galactose residues. To know whether B4GALT1 knockout alters sialylation of ITGB1 and ITGA6, we performed lectin pull-down assays of integrins using Maackia amurensis Lectin (MAL)-I, MAL-II, or (Sambucus nigra lectin) SNA. The results showed that B4GALT1 knockout did not significantly affect MAL-II or SNA binding to ITGB1 (Supplementary Fig. S9A). In contrast, the B4GALT1 knockout decreased MAL-I binding to ITGB1, suggesting that B4GALT1 may influence the decoration of α2,3 sialic acids on ITGB1. B4GALT1 knockout only slightly decreased MAL-I and SNA binding to ITGA6, indicating that B4GALT1 may influence the decoration of α2,3 and α2,6 sialic acids on ITGA6 (Supplementary Fig. S9B).

### Integrins β1 and α6 play a key role in HCC migration and invasion induced by B4GALT1 knockdown or knockout

**Fig. 6:**
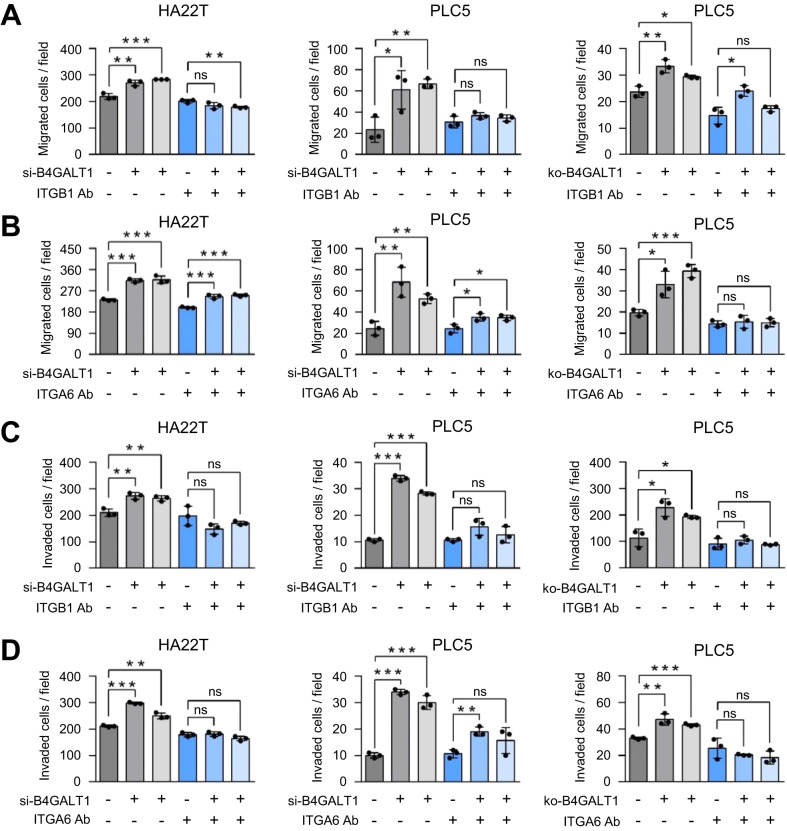
Blockade of integrin β1 or integrin α6 inhibits migration and invasion promoted by B4GALT1 knockdown or knockout in HCC cells. **A** Transwell migration assays showing the effect of anti-integrin β1 antibody. The increased cell migration increased by B4GALT1 siRNA in HA22T and PLC5 cells, or B4GALT1 knockout (ko) in PLC5 cells was significantly blocked by an anti-integrin β1 (ITGB1) antibody. Two different B4GALT1 siRNAs and one non-targeting control siRNA were used for HA22T and PLC5 cells. Two independent clones of B4GALT1 knockout PLC5 cells and wild-type PLC5 cells were used. Mouse IgG was used as control. **B** Transwell migration assays showing the effect of anti-integrin α6 antibody. The increased cell migration by B4GALT1 siRNA in HA22T and PLC5 cells, or B4GALT1 knockout (ko) in PLC5 cells was significantly blocked by anti-integrin α6 (ITGA6) antibody. **C** Matrigel invasion assays showing the effect of anti-integrin β1 antibody. The increased cell invasion increased by B4GALT1 siRNA in HA22T and PLC5 cells, or B4GALT1 knockout (ko) in PLC5 cells was significantly blocked by anti-ITGB1 antibody. **D** Matrigel invasion assays showing the effect of anti-integrin α6 antibody. The increased cell invasion increased by B4GALT1 siRNA in HA22T and PLC5 cells, or B4GALT1 knockout (ko) in PLC5 cells was significantly blocked by anti-ITGA6 antibody. Representative results from three independent experiments are shown. Data are presented as the mean ± SD. *P < 0.05; **P < 0.01; ***P < 0.001, Student’s *t*-test.

It has been demonstrated that integrin α6β1 is the main laminin receptor in HCC [9]. Moreover, we found that integrins β1 and α6 are the major B4GALT1 protein substrates in HCC cells. To evaluate whether integrins β1 and α6 are indeed involved in B4GALT1-regulated migration and invasion, blocking antibodies against integrin β1 and α6 were incubated with B4GALT1 knockdown or knockout HCC cells and then cells were subjected to transwell migration and Matrigel invasion assays. Representative images from the assays were shown in Supplementary Fig. S10. The results showed that the increased cell migration by B4GALT1 knockdown in HA22T and PLC5 cells or B4GALT1 knockout in PLC5 cells was inhibited by an anti-integrin β1 antibody (Fig. 6A) or an anti-integrin α6 antibody (Fig. 6B). Moreover, the increased cell invasion by B4GALT1 knockdown or knockout was also inhibited by the blocking antibodies for integrin β1 (Fig. 6C) and integrin α6 (Fig. 6D). These results suggest that integrins β1 and α6 are involved in the invasive behaviors induced by B4GALT1 downexpression in HCC cells.

Because receptor tyrosine kinases (RTKs) have been reported to promote HCC progression [35] and we found that glycosylation of insulin receptor can be modified by B4GALT1 (Supplementary Table S1), we analyzed whether B4GALT1 could regulate RTK activity using phospho-RTK array analysis. Our data showed that B4GALT1 knockdown did not evidently alter 58 phospho-RTK levels and their downstream signaling molecules p-AKT and p-ERK in PLC5 cells (Supplementary Fig. S11), suggesting a minor role of RTKs in the B4GALT1-regulated invasiveness of HCC cells. This result further emphasizes the significance of integrins β1 and α6 in B4GALT1-regulated invasiveness of HCC cells.

## Discussion

**Fig. 7:**
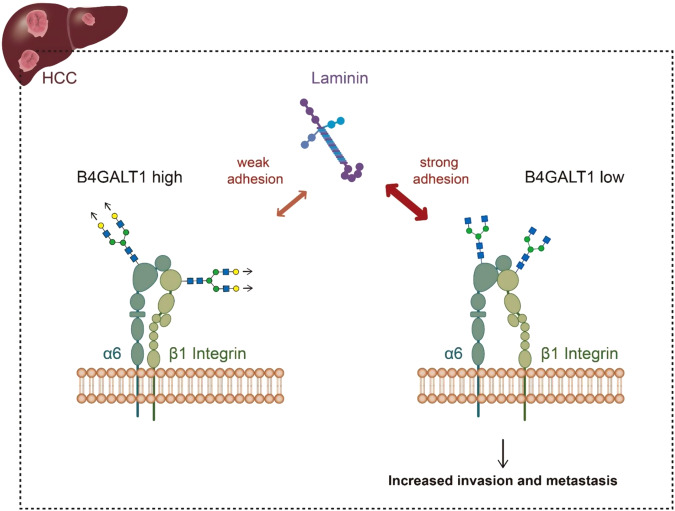
A schematic diagram illustrating the proposed mechanism by which B4GALT1 regulates HCC invasiveness. Downregulation of B4GALT1 promotes the invasive behaviors of HCC through the alteration of N-glycosylation and function of the laminin receptor, integrin α6β1.

Although B4GALT1 is the main β1,4-galactosyltransferase and is highly expressed in the liver, its expression and functional role in HCC remain unclear. Several reports have shown that B4GALT1 promotes malignant phenotypes and is associated with poor survival in many cancer types. Unexpectedly, we observed that B4GALT1 expression was downregulated in HCC and low B4GALT1 expression was associated with vascular invasion and poor overall survival of patients with HCC. In line with the clinical associations, silencing or loss of B4GALT1 expression promoted the invasiveness of HCC cells in vitro and enhanced lung metastasis in vivo. Mechanistic investigation indicated that downregulation of B4GALT1 enhanced HCC cell adhesion to laminin and integrins α6 and β1, subunits of the laminin receptor, were the main protein substrates of B4GALT1 to mediate the migration and invasion mediated by B4GALT1 knockdown or knockout (Fig. 7).

The correlation between B4GALT1 expression and malignant behaviors differs across various cancers. A high expression of B4GALT1 in tumors was associated with a poor prognosis in patients with pancreatic cancer, and B4GALT1 overexpression promoted cell migration and invasion [27]. B4GALT1 expression predicted a poor prognosis in muscle-invasive bladder cancer patients [26]. High B4GALT1 expression was associated with disease-free survival of patients with glioblastoma, and B4GALT1 knockdown increased apoptosis and autophagy [29]. By contrast, we found that low B4GALT1 expression was associated with a poor prognosis of HCC patients and enhanced cell invasive properties. The opposite role of B4GALT1 could be attributed to the existence of different B4GALT1 protein substrates in different cancer types.

A previous study showed that B4GALT1 was upregulated in HCC, and plays a positive role in HBx-induced hepatoma cell growth depending on its glycosylation activity [36]. Our results showed that B4GALT1 was downregulated in HCC. This discordance may result from whether the HCC tissues are infected with HBV since B4GALT1 is demonstrated to be a direct target gene of Hbx. With respect to the effect of B4GALT1 on cell growth, the previous study showed that B4GALT1 overexpression increased HepG2, Huh-7, and SMMC7721 cell growth. Our results showed that B4GALT1 knockdown slightly decreased PLC5 and SNU387, but increased HA22T cell growth. These findings suggest that the effect of B4GALT1 on HCC cell growth is cell-context dependent. Interestingly, our data consistently showed that silencing of B4GALT1 increased the invasive behavior of PLC5 and SNU387, and HA22T cells.

We systematically identified protein substrates of B4GALT1 using GSL-II lectin pulldown followed by mass spectrometry. To analyze the effect of B4GALT1 knockout on glycan structures in HCC cells, we used a panel of Gal-binding lectins, LEL, RCA-I, ECL, and PNA, as well as a GlcNAc-binding lectin GSL-II. Our data showed that B4GALT1 knockout did not significantly alter the binding of Gal-binding lectins to HCC cells. In contrast, GSL-II binding was dramatically increased. Therefore, GSL-II lectin is an ideal tool to identify B4GALT1 protein substrates. Proteomic analysis identified integrin β1 and integrin α6 as top 1 and 3 glycoproteins whose glycans were altered by B4GALT1. This is consistent with that integrin β1 was a protein substrate of mouse B4galt1 in megakaryocyte [22]. Interestingly, we are the first to show that the α subunit of integrin, α6 in this case, was also galactosylated by B4GALT1. Integrins have been well demonstrated to control cell adhesion, migration, and invasion [37]. In good agreement with the function of integrins, B4GALT1 can regulate cell adhesion to laminin and is associated with vascular invasion in HCC tumors. In addition to integrins, we identified other glycoproteins regulated by B4GALT1, such as neutral amino acid transporter (SLC1A5), insulin receptor (INSR), and growth/differentiation factor 15 (GDF15), which have been reported to modulate cancer malignant phenotypes [38–40]. However, our data from phospho-RTK array analysis showed that B4GALT1 knockdown did not evidently alter INSR phosphorylation. Moreover, blocking antibodies against integrin β1 and integrin α6 blocked most of the increased migration and invasion induced by B4GALT1 knockdown or knockout. These results therefore suggest that integrins β1 and α6 play a key role in B4GALT1-regulated invasiveness of HCC cells. It is still interesting to investigate whether B4GALT1 can modulate other biological functions through the above-mentioned glycoproteins.

Our data showed that silencing B4GALT1 enhances cell-laminin adhesion, migration, and invasion, which is dependent on integrin β1 and integrin α6. It has been reported that glycosylation contributes to the structural and functional role of integrins, and N-glycans have been demonstrated to be crucial for the conformation and stability of integrins, as well as the interaction with their ligands [41, 42]. Moreover, N-glycosylation of the I-like domain of the integrin β1 is essential to both the heterodimer formation and biological function of the subunit [43]. Therefore, our proposed mechanism by which silencing B4GALT1 promotes cell invasiveness is that low B4GALT1 expression alters N-glycans on integrin β1 and integrin α6, induces a more active conformation of integrin α6β1 heterodimer to enhance cell-laminin adhesion, and in turn increases cell migration and invasion in HCC cells.

We found that B4GALT1 knockout leads to exposure of GlcNAc on integrins and removal of GlcNAc using N-acetylglucosaminidase decreased cell adhesion to laminin, suggesting that GlcNAc plays a role in mediating cell migration and invasion probably through the alteration of integrin conformation. It has been reported that N-acetylglucosaminyltransferase (MGAT) family enzymes transfer GlcNAc sugars to N-glycans [44]. Therefore, it is reasonable to expect that MGAT enzymes could also regulate integrin glycosylation and activities to induce cell migration and invasion.

The interaction between tumor cells and laminins mediated by laminin-binding integrins is critical for tumor invasion and metastasis [45]. It has been reported that integrins α6 and β1 are overexpressed in HCC compared with normal liver and integrin α6β1 is the main laminin receptor in HCC [9, 46, 47]. Several lines of evidence showed that targeting integrin α6 or β1 was able to suppress HCC invasion and metastasis [45, 47–49]. Our data showed that low B4GALT1 enhanced HCC cell migration and invasion, which can be blocked by an antibody against integrin α6 or β1. Increasing numbers of integrin-targeted drugs with diverse effects are being developed [50, 51]. Although integrins have been well demonstrated to play a crucial role in tumor progression, these drugs only exhibit limited efficacy to cancer patients [52]. Therefore, discovering new strategies for integrin-targeted therapy is urgently needed. Further investigation warrants to test whether HCC patients with low B4GALT1 expression can benefit from antagonists of integrin α6 or β1.

Analysis of human samples showed that B4GALT1 was reduced in HCC and low B4GALT1 was associated with vascular invasion and poor prognosis of patients with HCC. Consistent with clinical findings, silencing or loss of B4GALT1 promoted HCC cell invasiveness in vitro and in vivo. Mechanistically, our data suggest that low B4GALT1 expression promotes HCC cell invasiveness primarily by modifying the N-glycans and functions of integrins α6 and β1. This study also emphasizes that B4GALT1-mediated galactosylation is a critical regulatory factor for integrin-related diseases.

## Materials and methods

### Clinical samples

The HCC tissues were collected from 78 patients who agreed to donate their clinical samples at our hospital between 2005 and 2010. This study complies with the Declaration of Helsinki and was performed according to the approval of the Institutional Review Board of the hospital (Approval No. 201712206RINC). Written consent was obtained from the patients. Information on the subjects is provided in Table 1.

### Immunohistochemistry (IHC)

The collected tissue slides were incubated with B4GALT1 polyclonal antibody (1:100; Abnova, Taipei, Taiwan) at 4 °C for 16 h. Thereafter, B4GALT1 protein expression was detected using the UltraVision Quanto Detection System (Thermo Scientific, Cheshire, UK). Further, the IHC staining assessment was independently conducted by two pathologists, who were blinded to the patient outcomes.

### Cell lines and cell culture

HCC cell lines, SKHEP1 (RRID: CVCL_0525), HepG2 (RRID: CVCL_0027), SNU387 ((RRID: CVCL_0250), and Huh7 (RRID: CVCL_0336) were purchased from Bioresource Collection and Research Center (Hsinchu, Taiwan), while the cell lines Hep3B (RRID: CVCL_0326), HA22T (RRID: CVCL_7046), and PLC5 (RRID: CVCL_0485) were obtained from American Type Culture Collection (Manassas, VA, USA). All the cell lines were maintained in Dulbecco’s modified Eagle’s medium (DMEM) (Thermo Fisher Scientific, Grand Island, NY, USA), which was supplemented with 10% Fetal Bovine Serum (FBS) (Thermo Fisher Scientific), 100 IU/mL penicillin, and 100 μg/mL streptomycin (Thermo Fisher Scientific). The culturing of the cells was performed in a humidified tissue culture incubator with a 5% CO_2_ atmosphere at 37 °C. All human cell lines have been authenticated using short tandem repeat profiling within three years. All experiments were performed with mycoplasma-free cells.

### Transfection and plasmid construction

To overexpress B4GALT1, pcDNA3.1/B4GALT1 plasmid was constructed and cells were transfected using Lipofectamine 3000 (Invitrogen, Carlsbad, CA, USA) as previously described. pcDNA3.1/myc-His (Invitrogen) was used as a control. The insert was confirmed via DNA sequencing. To transiently knock down B4GALT1, three independent siRNA oligonucleotides against B4GALT1 and negative control siRNAs with medium GC content were synthesized by Invitrogen. The siRNAs against B4GALT1 were si-B4GALT1-1: 5′-GAGGCAUGUCUAUAUCUCGCCCAAA-3′, si-B4GALT1-2: 5′-GAAGGACUAUGACUACACCUGCUUU-3′, and si-B4GALT1-3: 5′-CAACAGUUUCUAACCAUCAAUGGAU-3′. Further, for B4GALT1 knockdown, cells were transfected with 20 nmol of siRNA using Lipofectamine RNAiMAX (Invitrogen) for 2 days.

For stable B4GALT1 knockdown, the shB4GALT1/pLKO (TRCN34839) plasmid and non-targeting pLKO (TRC025) plasmid were purchased from National RNAi Core Facility (Academia Sinica, Taipei, Taiwan). Thereafter, short hairpin RNA (shRNA) plasmids were transfected for 48 h and selected using 500 ng/mL of puromycin for 10 days. The stable knockdown of B4GALT1 was then confirmed via western blot analysis.

### B4GALT1 knockout in PLC5 cells using the CRISPR/Cas9 system

The CRISPR/Cas9 system was used for B4GALT1 knockout in PLC5 cells. Further, a small guide (sg) RNA for targeting B4GALT1 was designed according to the CRISPR database prediction (http://crispr.mit.edu/). The target sequence of sgB4GALT1 was 5′-CCTGTACGCATTATGGTCATTCA-3′. The success of the B4GALT1 knockout in the genome was confirmed via DNA sequencing.

### Mass spectrometric analysis

LC-MS/MS analysis was performed on Orbitrap Fusion Lumos Tribrid quadrupole-ion trap-Orbitrap mass spectrometer (Thermo Fisher Scientific, San Jose, CA). Peptides were separated on Ultimate system 3000 nanoLC system (Thermo Fisher Scientific, Bremen, Germany), which was connected to the mass spectrometer. Peptide mixtures were loaded onto a 75 μm ID, 25 cm length C18 Acclaim PepMap NanoLC column (Thermo Scientific, San Jose, CA, USA) packed with 2 μm particles with a pore of 100 Å. Mobile phase A was performed using 0.1% formic acid in water, and mobile phase B was composed of 100% acetonitrile with 0.1% formic acid. A segmented gradient in 50 min from 2% to 40% solvent B was used with flow rate of 300 nL/min. Mass spectrometric analysis was performed in a data-dependent mode with Full-MS (a resolution of 120,000 at m/z = 200, AGC target 5e5, and maximum injection time of 50 msec). It was followed by high-energy collision-activated dissociation (HCD)-MS/MS of the top 15 most intense ions in 3 seconds. HCD-MS/MS was used to fragment multiply charged ions within a 1.4 Da isolation window (resolution of 15,000) at a normalized collision energy of 32. AGC target 4e4 was set for MS/MS analysis with previously selected ions dynamically excluded for 60 seconds.

For protein identification, the raw MS/MS data were searched using the Mascot and SEQUEST search algorithm via the Proteome Discoverer (PD) package (version 2.3, Thermo Scientific). The search parameters were set as follows: peptide mass tolerance, 10 p.p.m.; MS/MS ion mass tolerance, 0.02 Da. Peptides were filtered based on a 1% FDR.

### Antibodies and reagents

Antibody against B4GALT1 was obtained from Abnova (Cat#PAB20512), while that against integrin β1 (CD29) was purchased from BD Transduction Laboratories (Cat#610468). Further, antibody against integrin **α**6 was obtained from Cell Signaling Technology Inc. (Cat#3750), while that against GAPDH was purchased from Meridian Life Science. (Cat#H86504M). Functional blocking antibodies for integrin β1 (P4C10) were obtained from Merck Millipore (Cat#MAB1987Z). Further, functional blocking antibodies for integrin **α**6 (CD49f) were purchased from Invitrogen (Cat#12-0495-82), and human collagen I, human collagen IV, human fibronectin, laminin, or bovine serum albumin (BSA) were purchased from Sigma Aldrich (St Louis, MO, USA). GSL-II-FITC, LEL-FITC, RCA-I-FITC, ECL-FITC, MAL-II-FITC, and PNA-FITC were purchased from Vector Laboratories (Burlingame, CA, USA).

### Western blot analysis

Proteins were separated via SDS-PAGE, and then transferred onto PVDF membranes. The membrane blots then were blocked with 5% milk in TBST for 1 h at room temperature, and thereafter, incubated with primary antibodies overnight at 4 °C. The blots were then incubated with a corresponding secondary antibody conjugated with horseradish peroxidase, and signals were detected using ECL reagents and X-ray films.

### Experimental metastasis model in NOD/SCID mice

Female NOD/SCID mice (4 weeks old; LASCO, Taipei, Taiwan) were injected via the tail vein with 1 × 10^6^ HCC cells. After 60 days, the mice were sacrificed and metastatic nodules were visualized. Specifically, after sacrifice, the lungs were paraffin-embedded for hematoxylin and eosin (H&E) staining. All mice used in this study were maintained under specific pathogen-free conditions and housed in pathogen-free facilities in a 12 h light/dark cycle with ad libitum access to food and water. This animal study was reviewed and approved by the Institutional Animal Care and Use Committee (IACUC) of National Taiwan University College of Medicine (Approval No. 20170405).

### MTT assay

HCC cells in 100 μL of complete DMEM were seeded in 96-well plates at a cell density of 3 × 10^3^ cells per well. Thereafter, 10 μL of 5 mg/ml 3-(4,5-dimethyl-2-thiazolyl)-2,5-diphenyl-2H-tetrazolium bromide solution (MTT, Sigma) were added to each well for the indicated times followed by incubation at 37 °C for 4 h, after which 100 μL of 10% SDS in 0.01 N HCl was added to dissolve the MTT formazan crystals. The resultant optical density was then measured spectrophotometrically at dual wavelengths (550 and 630 nm).

### Transwell migration and Matrigel invasion assays

Transwell migration assays were performed using 6.5-mm polycarbonate transwell filters with an 8-μm pore size (Corning Costar Corp., Corning, NY, USA). In brief, approximately 3 × 10^4^ cells (HA22T) or 2 × 10^5^ cells (PLC5) in 200 μL serum-free DMEM were seeded into the upper surface of the transwell chamber, while 500 μL of 10% FBS in complete DMEM was loaded into the lower chamber of 24-well plates. Thereafter, BioCoat Matrigel invasion chambers (BD PharMingen, San Diego, CA, USA) were used for the cell invasion assay. The cells were allowed to migrate toward the transwell chamber or invade the Matrigel for 24 h. The migrated and invaded cells we then fixed with 100% methanol and stained with 0.5% (w/v) crystal violet (Sigma-Aldrich). Thereafter, the numbers of migrated and invaded cells per field were counted in at least three independent experiments (mean ± SD). For integrin functional blocking experiments, 100 μg/ml of blocking antibodies against integrin β1, integrin **α**6, or control mouse IgG were treated for 30 min before seeding into the upper chamber of transwells. Cells were then subjected to migration and invasion assays.

### Flow cytometry

Cell surface protein expression was analyzed using the FACScan cytometer (BD PharMingen). In brief, HCC cells were detached using 5 mM EDTA and resuspended in 2% BSA/PBS. Thereafter, the cells were incubated with FITC-conjugated lectins at 1∶100 dilutions on ice for 30 min. Next, the cells were washed three times with ice-cold 2% BSA/PBS, and the fluorescence intensity of 1 × 10^5^ cells for each sample was analyzed.

### Lectin pull-down assay

For the lectin pull-down assay, cell lysates (1 mg) were incubated with *Griffonia Simplicifolia* Lectin II (GSL-II) biotinylated (Vector Laboratories) at 4 °C for 16 h. The next day, the sample was incubated with streptavidin beads (Vector Laboratories) for 2 h at room temperature. Finally, the pull-down proteins were analyzed via western blot analysis.

### Cell adhesion assay

Cell adhesion assays were performed as previously reported [17]. In brief, 96-well plates were coated with BSA, fibronectin, laminin, collagen I, or collagen IV at concentrations of 2.5 μg/mL in PBS at 37 °C for 16 h, and then blocked with 1% BSA in PBS at 37 °C for 4 h. Next, the HCC cells were detached using 5 mM EDTA and 2 × 10^4^ cells in 100 μL serum-free DMEM were seeded and allowed to attach for 30 min at 37 °C in a humidified 5%-CO_2_ incubator. Finally, the number of adherent cells in three wells were determined using an inverted microscope.

### Statistical analyses

Statistical analyses were performed using GraphPad Prism software version 7 (GraphPad Software Inc., San Diego, CA, USA). The correlations between B4GALT1 expression and the clinicopathological characteristics of HCC were tested by performing chi-square tests. Further, survival curves were constructed using the Kaplan–Meier method and the Student’s *t*-test was performed to compare differences between two groups of quantitative variables. Data were presented as means ± SD or number (percentage), and statistical significance was set at *p* < 0.05.

### Supplementary information

Supplementary Table

Supplementary Figures

## Data Availability

The data that support the findings of this study are available in the Supporting Information of this article (Figs. S1–S9 and Table S1).
