# Supplementary material for: Decreased B4GALT1 promotes hepatocellular carcinoma cell invasiveness by regulating the laminin-integrin pathway
Source: Oncogenesis. 2023 Oct 31;12(1):49. doi: 10.1038/s41389-023-00494-y (PMC10618527; doi:10.1038/s41389-023-00494-y)
Supplement: Supplementary file 1 — Supplementary Table [file 41389_2023_494_MOESM1_ESM.docx]

| **Supplementary Table S1. Glycoproteomic analysis reveals potential substrates of B4GALT1 in PLC5 cells** | | | | | | | |
| --- | --- | --- | --- | --- | --- | --- | --- |
| Rank | Gene | Accession | Protein name | MW [kDa] | calc. pI | Mascot score | Fold change* (GSL-II pulldown, KO/WT PLC5) |
| 1 | ITGB1 | P05556 | Integrin beta-1 | 88.4 | 5.39 | 534 | 24.52 |
| 2 | SLC1A5 | Q15758 | Neutral amino acid transporter B(0) | 56.6 | 5.48 | 170 | 9.84 |
| 3 | ITGA6 | P23229 | Integrin alpha-6 | 126.5 | 6.61 | 39 | 8.26 |
| 4 | CP | P00450 | Ceruloplasmin | 122.1 | 5.72 | 155 | 7.43 |
| 5 | SLC39A14 | Q15043 | Zinc transporter ZIP14 | 54.2 | 5.33 | 38 | 5.76 |
| 6 | LGALS3BP | Q08380 | Galectin-3-binding protein | 65.3 | 5.27 | 858 | 4.58 |
| 7 | INSR | P06213 | Insulin receptor | 156.2 | 6.2 | 246 | 3.41 |
| 8 | GDF15 | Q99988 | Growth/differentiation factor 15 | 34.1 | 9.66 | 207 | 2.65 |
| 9 | KDELR2 | P33947 | ER lumen protein-retaining receptor 2 | 24.4 | 8.72 | 92 | 2.11 |
| 10 | KDELR1 | P24390 | ER lumen protein-retaining receptor 1 | 24.5 | 8.62 | 94 | 2.00 |

* Only proteins with fold change ≥ 2 in the secretory pathway are listed. KO, B4GALT1 knockout; WT, wild type.
