# Supplementary material for: Decreased B4GALT1 promotes hepatocellular carcinoma cell invasiveness by regulating the laminin-integrin pathway
Source: Oncogenesis. 2023 Oct 31;12(1):49. doi: 10.1038/s41389-023-00494-y (PMC10618527; doi:10.1038/s41389-023-00494-y)
Supplement: Supplementary file 2 — Supplementary Figures [file 41389_2023_494_MOESM2_ESM.pdf]

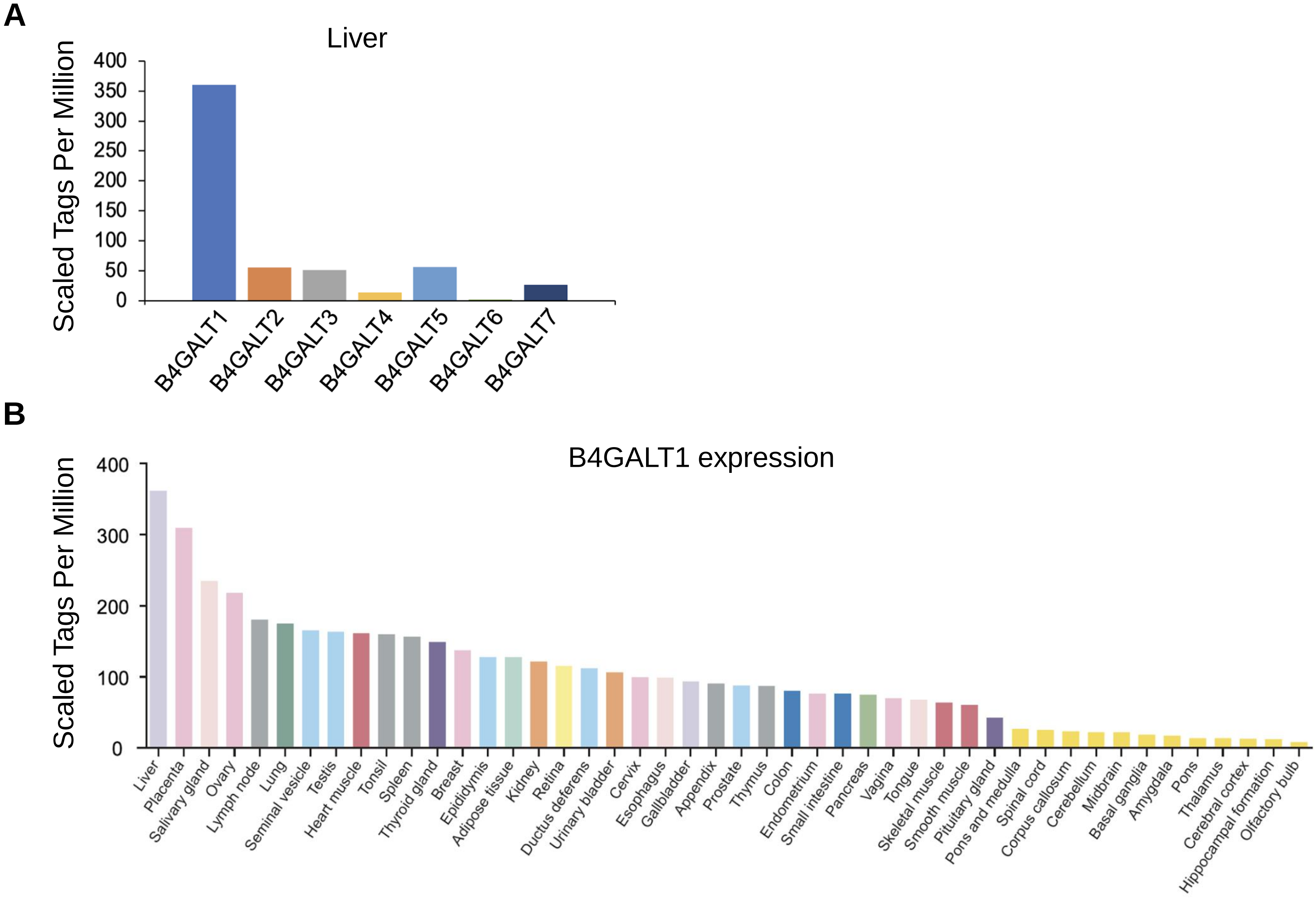

Supplementary Figure S1. *B4GALT1* is highly expressed in liver. A. Among seven members of the *B4GALT* family, *B4GALT1* is most highly expressed in liver. **B.** Among 46 human tissues, *B4GALT1* is most highly expressed in liver. Data were retrieved from the FANTOM5 dataset in The Human Protein Atlas (HPA).

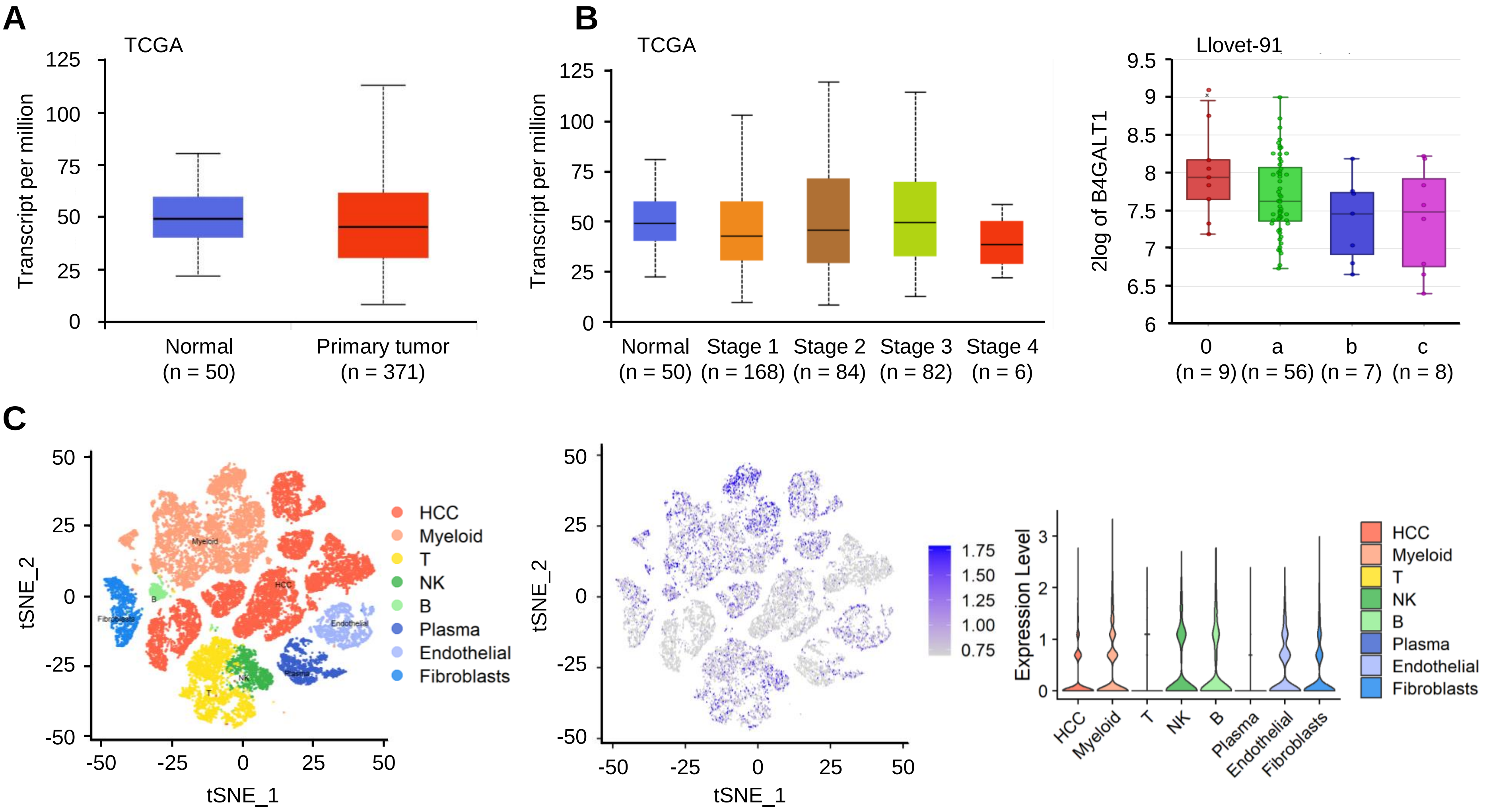

Supplementary Figure S2. B4GALT1 expression in normal and HCC tissues. A. *B4GALT1* mRNA expression between normal liver and hepatocellular carcinoma (HCC) tissues in the the Cancer Genome Atlas (TCGA) database analyzed using the UALCAN platform. **B.** Correlation between *B4GALT1* and HCC stages in two databases. Left panel, the TCGA database showing clinical pathological stages (stage1, stage2, stage3 and stage4) of HCC. Right panel, the Llovet-91 database showing the Barcelona Clinic Liver Cancer (BCLC) staging system of HCC (0-very early stage, a-early stage, b-intermediate stage and c-advanced stage). **C.** scRNA-seq profiling (dataset ID: EGAD00001006190) of *B4GALT1* in HCC and stromal cells. Left panel, the T-distributed Stochastic Neighbor Embedding (tSNE) plot of 8 cell clusters from 10 HCC tumors. Cells from different clusters are marked by colors. Middle panel, the plot showing the *B4GALT1* expression level in 8 cell clusters. Right panel, the violin plot showing relative *B4GALT1* expression levels in 8 cell clusters.

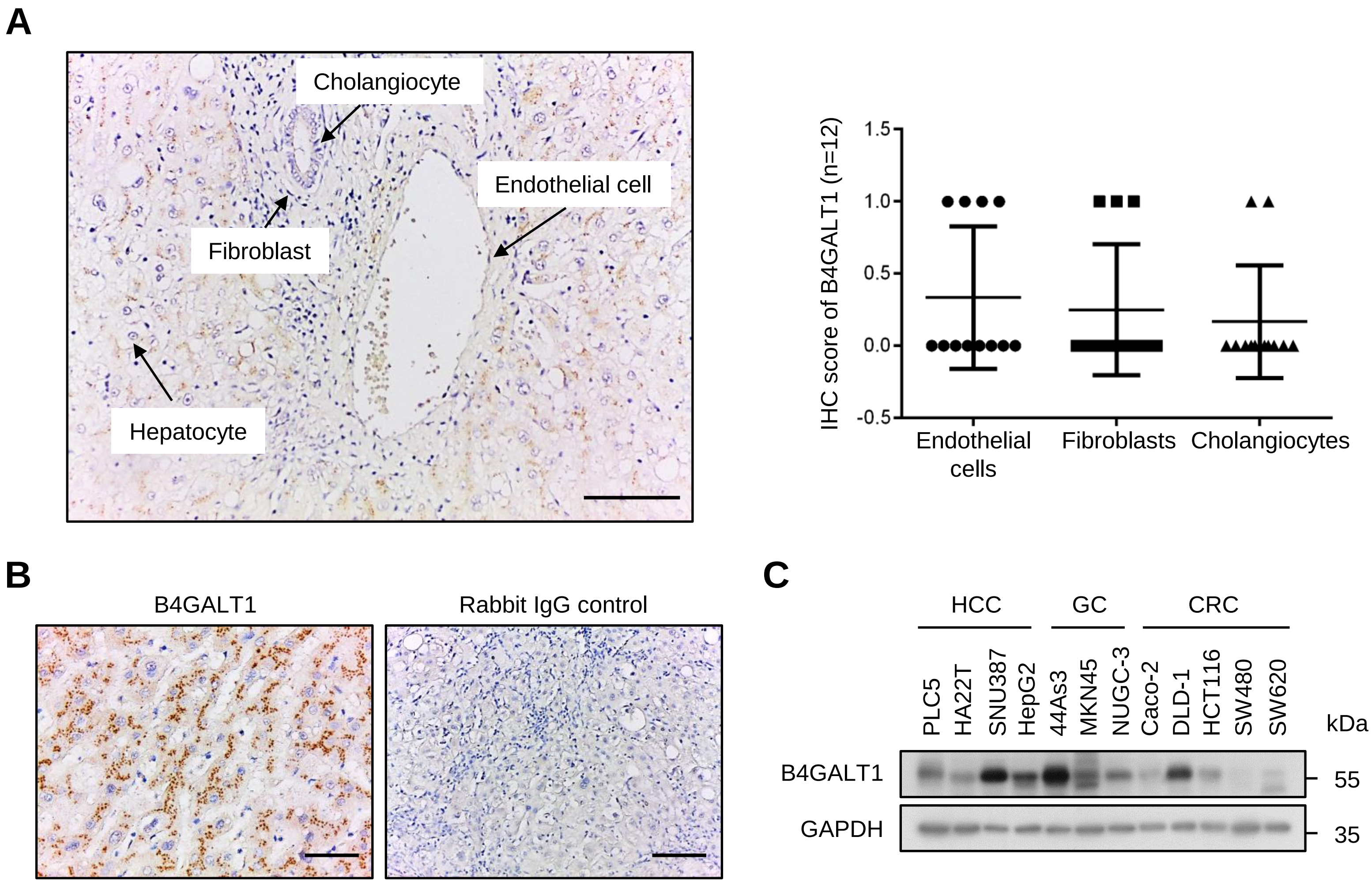

Supplementary Figure S3. Expression of B4GALT1 in liver stromal cells and in various cancer cell lines. **A.** B4GALT1 expression in liver stromal cells including endothelial cells, fibroblasts, and cholangiocytes. Left panel, representative image showing immunohistochemistry (IHC) of B4GALT1 in normal liver. Scale bar, 50 μm. Right panel, IHC score of B4GALT1 expression in stromal cells, as indicated. **B.** Specificity of anti-B4GALT1 antibody for IHC. Left panel, B4GALT1 staining in normal liver. Right panel, rabbit IgG control did not show any specific staining in liver and was used for the negative control. Scale bar, 50 μm. **C.** Western blot analysis of B4GALT1 in hepatocellular carcinoma (HCC), gastric cancer (GC) and colorectal cancer (CRC) cell lines, as indicated.

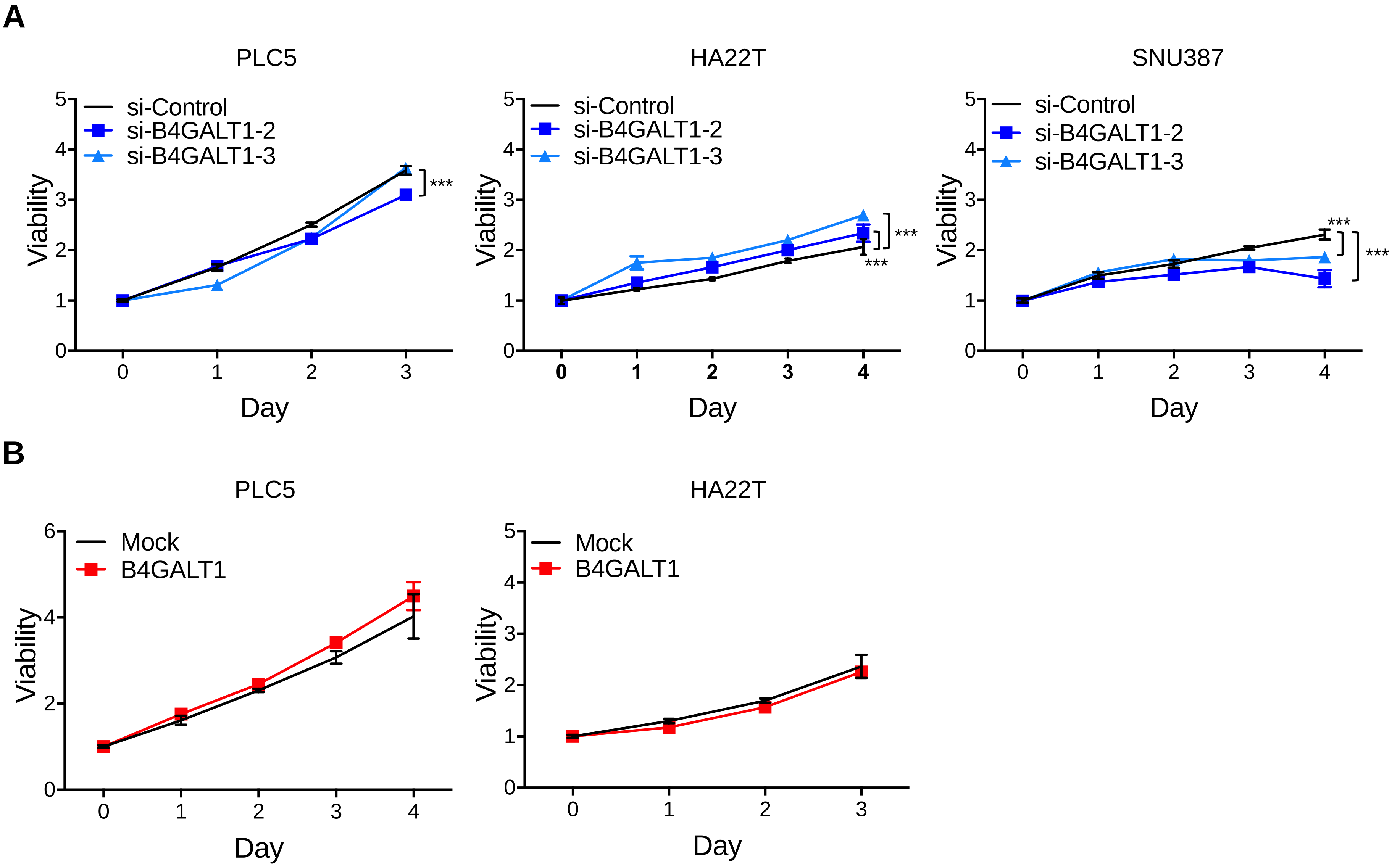

Supplementary Figure S4. MTT assays showing effects of B4GALT1 on HCC cell viability. A. Effects of B4GALT1 knockdown on HCC cells, as indicated. *** *P*<0.001, by Student's t-test. **B.** Effects of B4GALT1 overexpression on HCC cells, as indicated. . *** *P*<0.001, by Student's t-test.

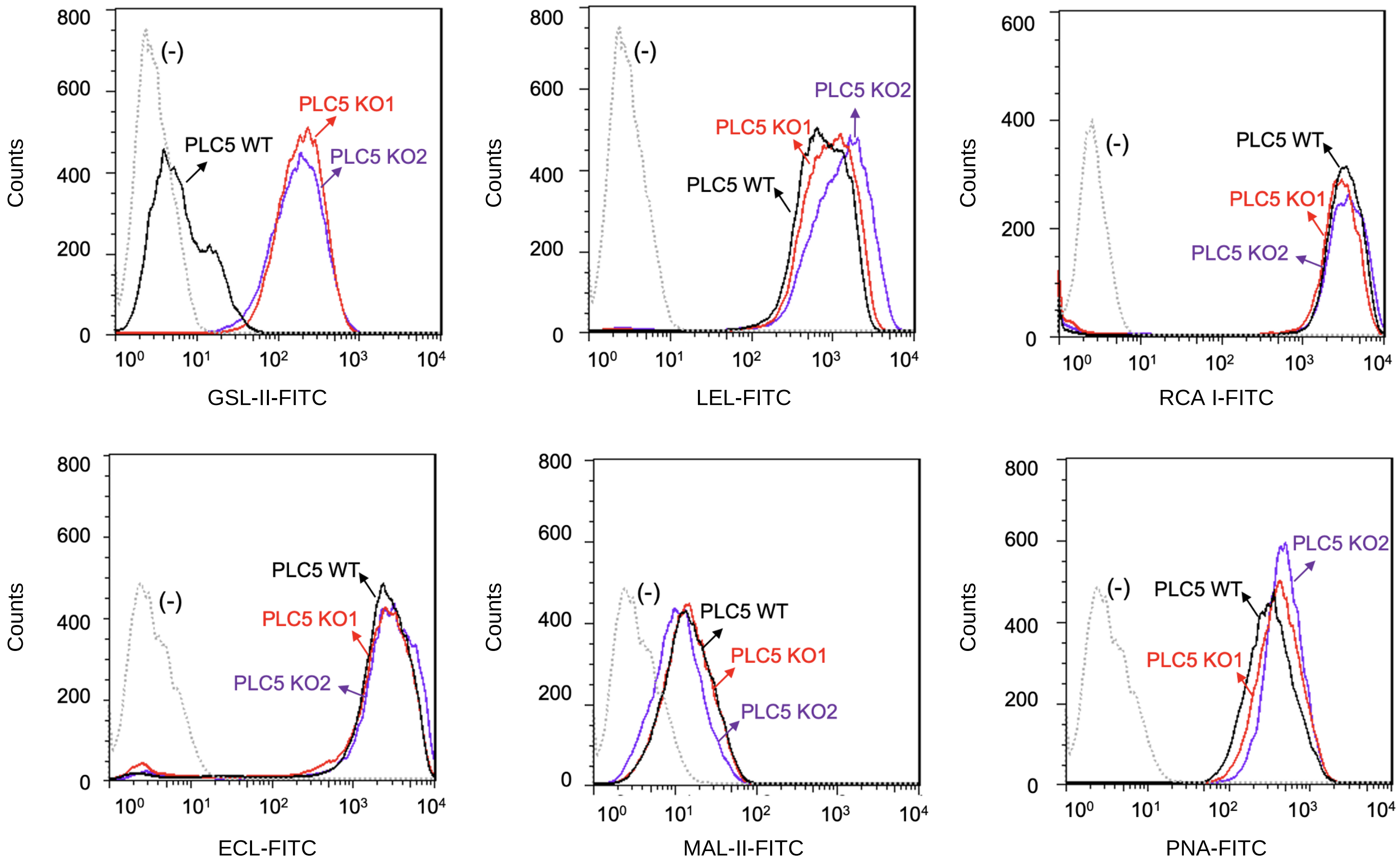

Supplementary Figure S5. Effects of B4GALT1 knockout on glycophenotypes in PLC5 cells. Glycan expression on the surface of B4GALT1 wild-type (WT) or knockout (KO) PLC5 cells (two clones: KO1 and KO2) was analyzed using flow cytometry with FITC-conjugated lectins, as indicated.

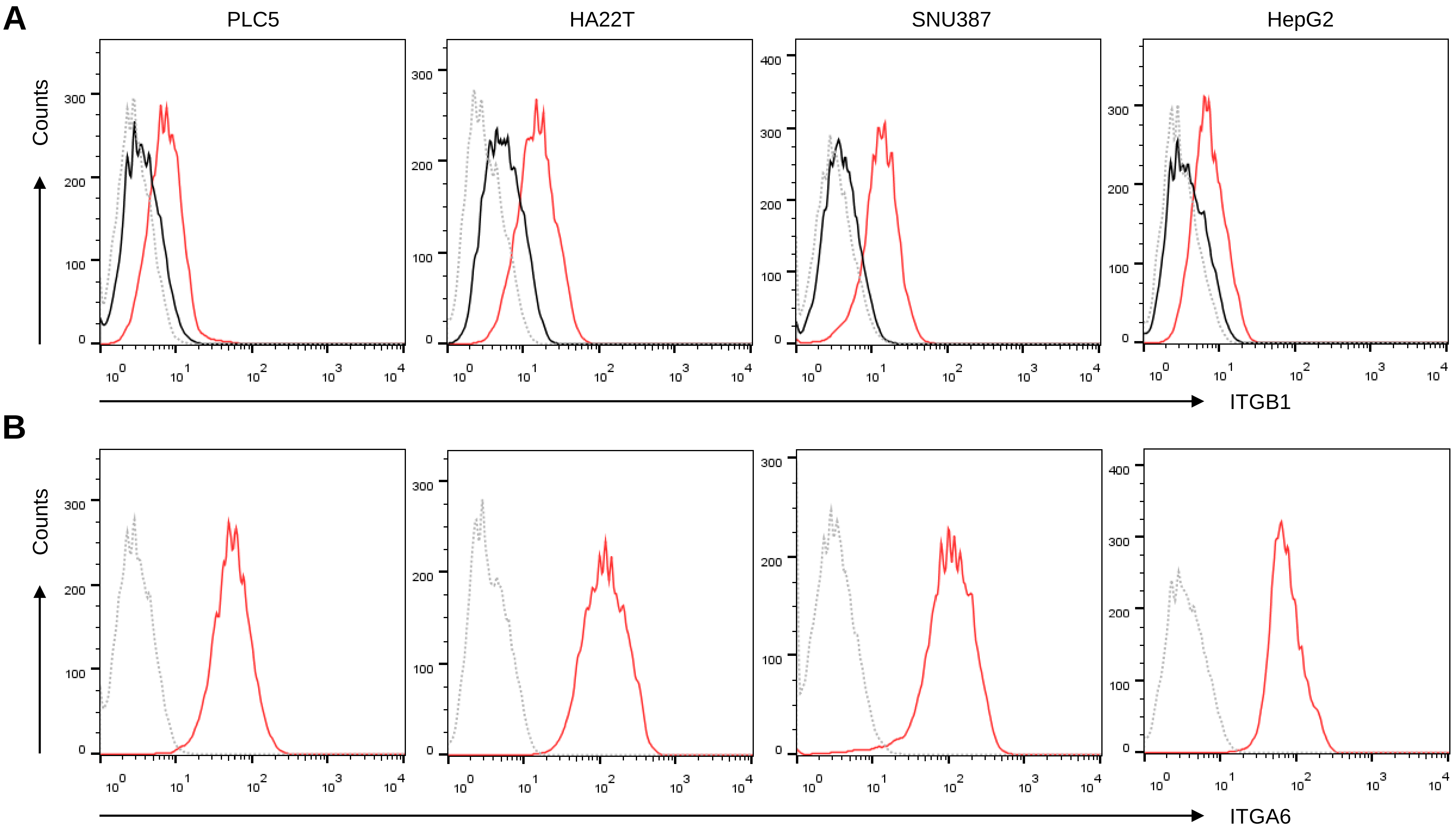

Supplementary Figure S6. Flow cytometric analysis of ITGB1 and ITGA6 expression on the cell surface of HCC cells. A. Flow cytometry of ITGB1 in HCC cells, as indicated. **B.** Flow cytometry of ITGA6. Dotted lines indicate cells stained with matched primary antibody. Black solid lines indicate cells stained with FITC-conjugated secondary antibody alone. Red lines indicate cells stained with an anti-integrin antibody. Anti-ITGB1 antibody is a purified antibody without any conjugation. Anti-ITGA6 antibody is PE-conjugated.

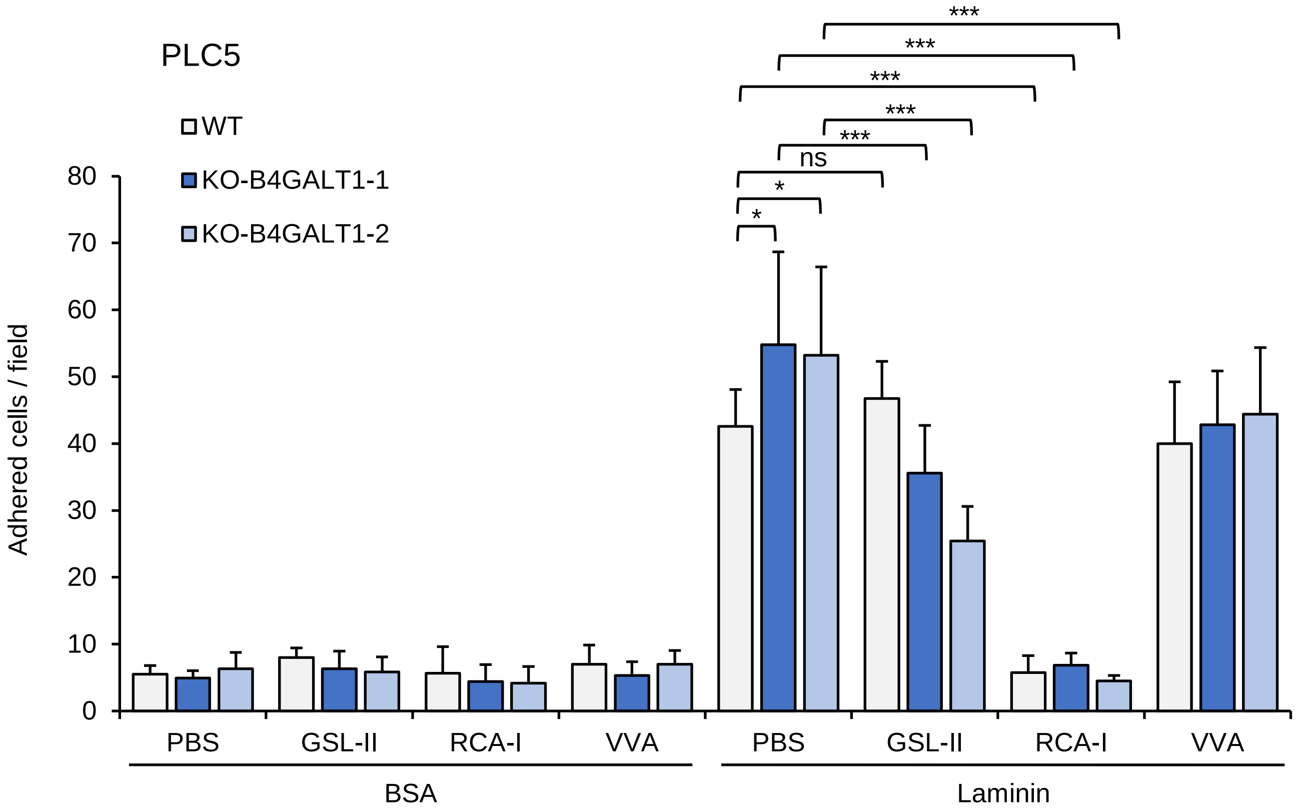

Supplementary Figure S7. Effects of GSL II, RCA I, and VVA lectin on cell-laminin adhesion. WT or B4GALT1 KO PLC5 cells were pre-treated with various lectins, as indicated, for 10 min and allowed to attach on laminin-coated plates for 30 min at 4°C. Adhered cells were counted under a microscope. BSA was used as a non-ECM protein control. Results are shown as means ± SD. * *P* < 0.05; *** *P* < 0.001; ns, not significant.

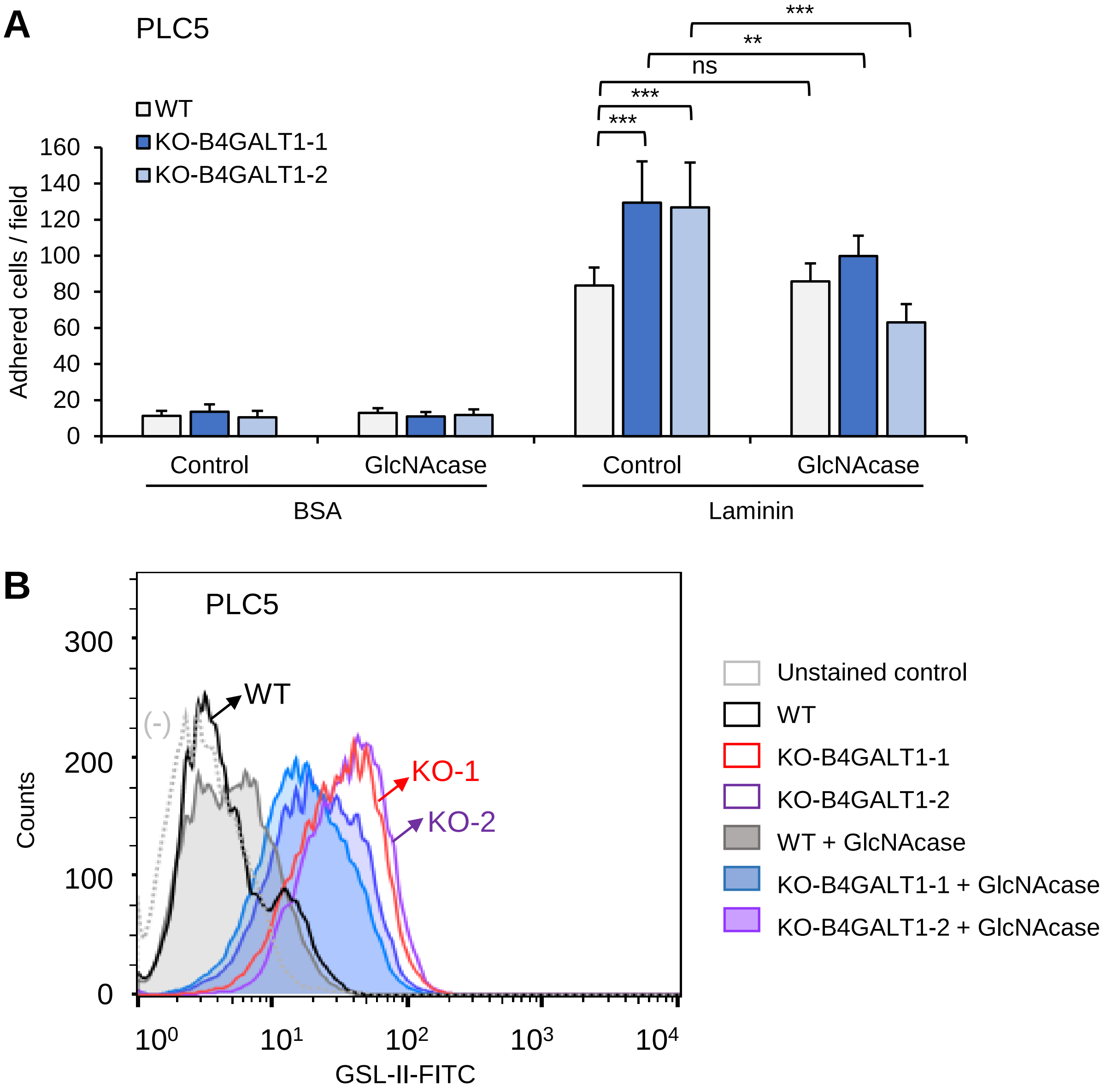

Supplementary Figure S8. Effects of N-acetylglucosaminidase on cell-laminin adhesion and GSL II binding. A. Impact of N-acetylglucosaminidase (GlcNAcase) on cell-laminin adhesion. Wild-type or B4GALT1 KO PLC5 cells were pre-treated with GlcNAcase or PBS control for 30 min and then plated into laminin-coated 96-well plates for 30 min. BSA was used for the control. Results are presented as the means ± SD. ** $P < 0.01$; *** $P < 0.001$; ns, not significant. **B.** Wild type (WT) and B4GALT1 KO cells were treated with GlcNAcase, and then GSL II binding was analyzed using flow cytometry.

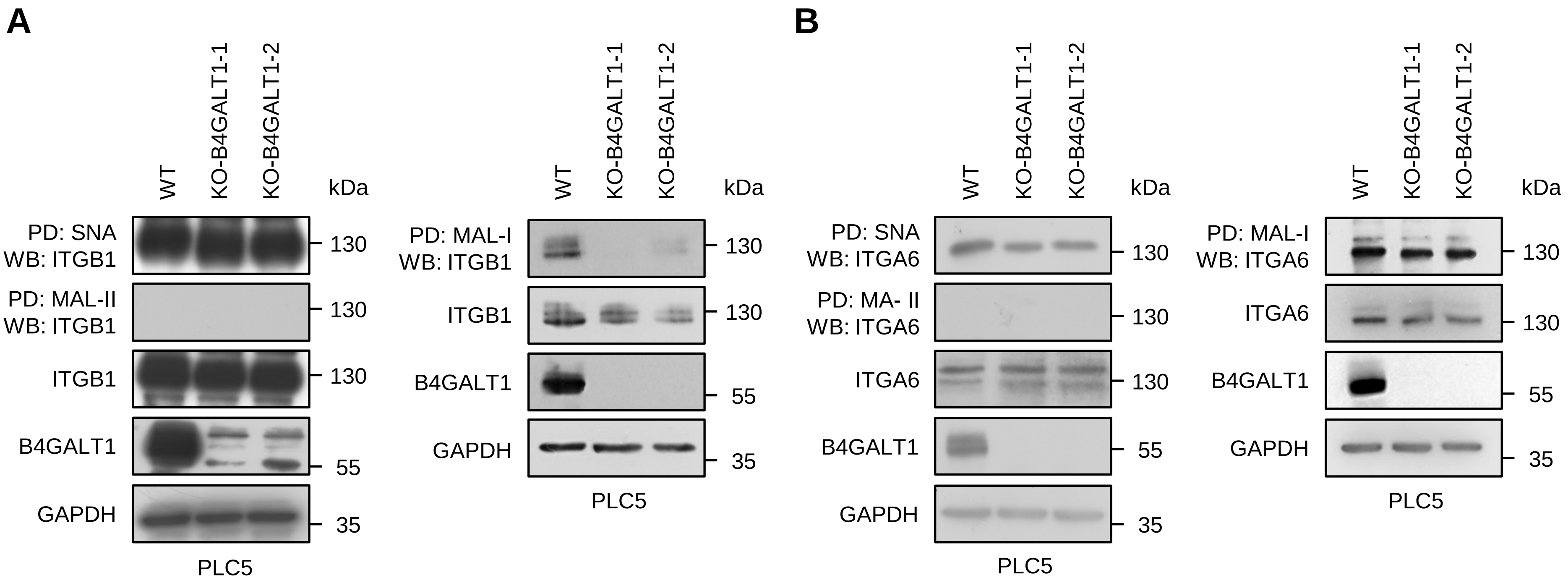

Supplementary Figure S9. Effects of B4GALT1 knockout on sialylation of ITGB1 and ITGA6 in PLC5 cells. **A.** Lectin pull-down assay of ITGB1. **B.** Lectin pull-down assay of ITGA6. Cellular proteins in WT or B4GALT1 KO PLC5 cells were pulled down (PD) with SNA, MAL I or MAL II lectin and then analyzed using Western blot (WB) analysis of integrins.

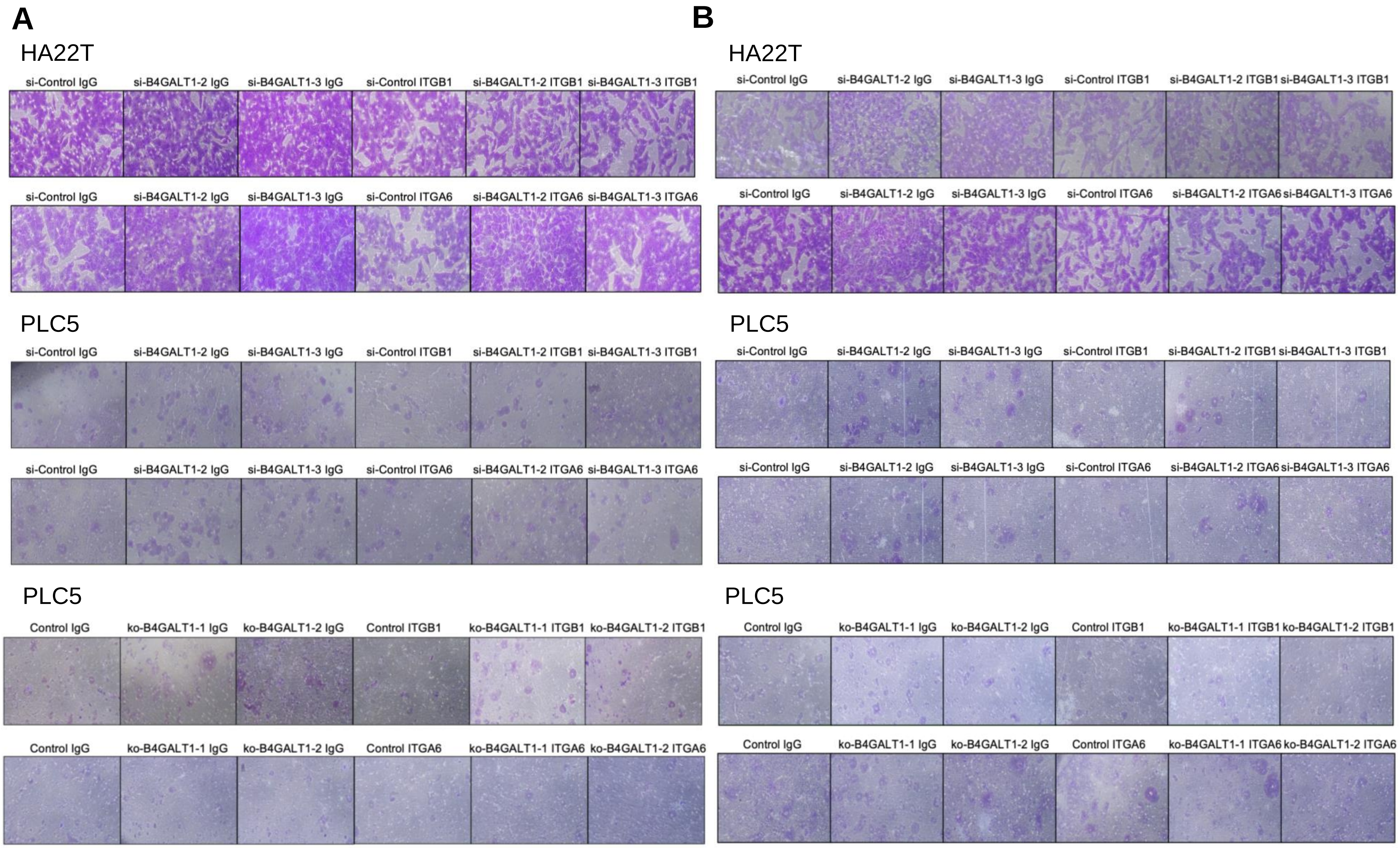

Supplementary Figure S10. Representative images of HCC cell migration and invasion blocked with an anti-integrin β1 or anti-integrin α6 antibody. A. Cell migration was analyzed using transwell migration assays. **B.** Cell invasion was analyzed using Matrigel invasion assays. To knock down B4GALT1 in HA22T and PLC5 cells, two independent siRNAs were used (si-B4GALT1-2, and si-B4GALT1-3), as indicated. To knock out B4GALT1 in PLC5 cells, two different clones were used (ko-B4GALT1-1 and ko-B4GALT1-2), as indicated. An anti-integrin β1 (ITGB1), anti-integrin α6 (ITGA6) antibody, or control IgG was added in the upper chamber, as indicated.

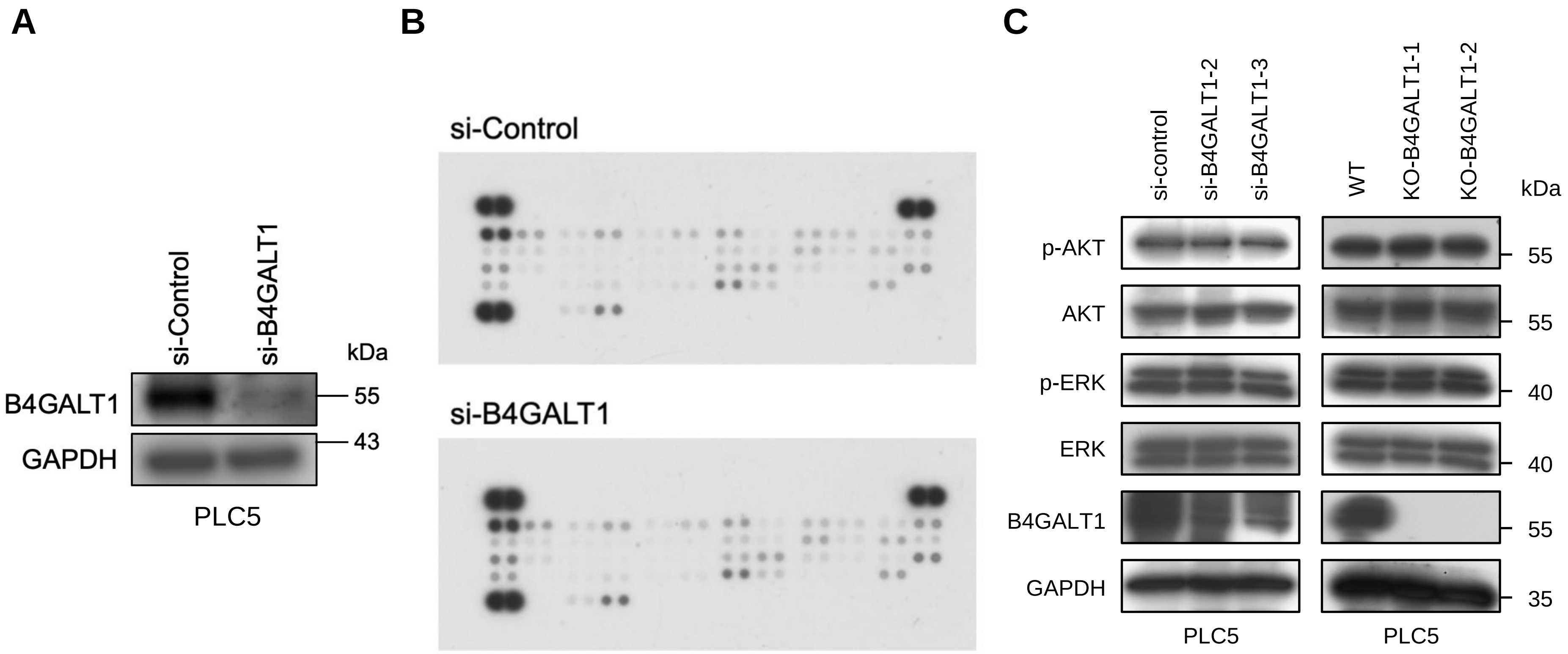

Supplementary Figure S11. B4GALT1 knockdown does not significantly change levels of phospho-RTKs. A. Western blots showing B4GALT1 knockdown with siRNA in PLC5 cells. GAPDH is an internal control. **B.** Phospho-RTK array analysis of PLC5 cells knocked down with control siRNA or B4GALT1 siRNA. **C.** Western blots showing effects of B4GALT1 knockdown or knockout on phosphorylation of AKT and ERK in PLC5 cells.
